# Bilirubin kinetics in preterm infants

**DOI:** 10.1038/s41372-026-02886-5

**Published:** 2026-08-31

**Authors:** Thomas Hegyi, Natasha Cordero, Amrryn Halari, Anna Petrova

**Affiliations:** The Division of Neonatology, Department of Pediatrics, Robert Wood Johnson Medical School, Rutgers University, New Brunswick, NJ USA

**Keywords:** Risk factors, Haematological diseases

## Abstract

**Background:**

Preterm infants are vulnerable to bilirubin neurotoxicity. We evaluated whether time-stratified transcutaneous bilirubin (TcB) and kinetic indices improve the prediction of the risk of phototherapy and escalation.

**Methods:**

In a cohort of preterm infants, only pre-phototherapy TcB values were analyzed. TcB values were grouped by postnatal age (≤24 h, 25–48 h, 49–72 h, ≥73 h), and sequential measurements were used to calculate bilirubin increase rates (RBIa, RBIb) and absolute percent change (APC%).

**Results:**

Infants requiring phototherapy had higher TcB levels within 72 h, particularly at ≤24 h and 25–48 h. Independent predictors included TcB (OR 2.11), gestational age (OR 0.37), and postnatal age (OR 0.30; AUC 0.827). Treated infants showed faster bilirubin accumulation, and early RBIa correlated with peak TcB (*r* = 0.334). APC% independently predicted proximity to the escalation threshold.

**Conclusions:**

Time-standardized TcB measurements and dynamic kinetic indices may facilitate earlier identification of preterm infants at increased risk for phototherapy and warrant prospective validation.

## Introduction

Preterm infants experience a higher risk of acute bilirubin encephalopathy (ABE) and bilirubin-associated neurotoxicity than term newborns [1]. This vulnerability stems from multiple compounding physiological factors, including reduced hepatic uridine diphosphate glucuronosyltransferase (UGT1A1) conjugation capacity, enhanced enterohepatic circulation, and increased blood–brain barrier permeability [2, 3]. Consequently, classical kernicterus and subtle ABE occur in preterm populations at total serum bilirubin (TSB) levels considered safe for mature neonates and occasionally present in very low-birth-weight infants without marked clinical jaundice [1, 4].

Current clinical guidelines focus exclusively on infants born at 35 or more weeks of gestation [5]. For these mature infants, tracking the velocity of bilirubin rise has been integrated into safety algorithms, in which increments exceeding 0.3 mg/dL/h on day 1 or 0.2 mg/dL/h on subsequent days serve as clinical triggers for intensified monitoring [5, 6]. This kinetic philosophy underscores that the directional velocity of bilirubin accumulation provides diagnostic utility that matches or exceeds that of a single, static snapshot [6–8]. Unfortunately, direct guidance and standardized predictive tools tailored to the unique kinetics of infants born at or before 35 weeks remain sparse, leading to reliance on single-threshold cross-sections [9, 10].

A profound methodological challenge has historically hindered the development of reliable models for preterm bilirubin. In real-world NICU settings, transcutaneous bilirubinometry (TcB) measurements are collected at heterogeneous, clinically driven timelines rather than rigid protocol-fixed intervals [11]. This structural irregularity prevents valid side-by-side longitudinal tracking. Furthermore, standard data models frequently commit an analytical error by failing to censor post-treatment values within the phototherapy cohort. Because phototherapy drives bilirubin levels sharply downward, including post-treatment data points introduces an artificial deflation of true disease burden, rendering subsequent statistical inferences invalid [12, 13].

To address these systemic gaps, this study presents a unified analysis that draws on a prospective cohort of preterm infants and employs dual analytical paradigms to rigorously characterize native bilirubin dynamics during the first week of life [14]. First, we apply a data reconstruction technique that assigns unstructured observations into standardized postnatal age bins to generate a robust multivariable prediction model for phototherapy requirements. Second, we trace sequential, unbinned measurement pairs to calculate precise rate-of-change indices, including the absolute rate of bilirubin increase (RBIa), the interval rate of increase (RBIb), and the absolute percent change (APC%), to determine whether future treatment candidates are kinetically distinct before standard intervention criteria are met. Finally, we explore how early dynamic velocities correlate with peak bilirubin values and an actionable look-ahead escalation-of-care threshold (ECT).

## Methods

### Study design and population

This study was a secondary analysis of a prospective observational cohort conducted in the neonatal intensive care unit at Bristol Myers Squibb Children’s Hospital, Robert Wood Johnson University Hospital, New Brunswick, New Jersey, between January 2023 and April 2025. The original cohort investigated the effects of race, postnatal age, and phototherapy on transcutaneous bilirubin measurements in preterm infants and has been reported previously [14]. The present analysis included all infants from the original cohort and addressed the distinct objective of characterizing bilirubin kinetics and evaluating predictors of subsequent phototherapy.

Preterm infants born at less than 36 weeks’ gestation who underwent serial transcutaneous bilirubin (TcB) measurements during the first week of life were eligible for inclusion. Gestational age was determined from the best obstetric estimate using early ultrasonography and the last menstrual period when available. Demographic and clinical information, including birth weight, sex, race and ethnicity, Coombs status, and clinical diagnoses, were obtained from the electronic medical record.

The study cohort comprised 113 infants, including 28 who received phototherapy and 85 who did not. For infants who underwent phototherapy, only TcB measurements obtained before initiation of the first course of phototherapy were included in the analyses. No readmitted infants were included in the study. This approach was used to characterize native bilirubin kinetics and to avoid confounding by the bilirubin-lowering effects of phototherapy.

The study was approved by the Rutgers Robert Wood Johnson Medical School Institutional Review Board (Pro2023000044). Written informed parental permission was obtained before enrollment. The study was conducted in accordance with the principles of the Declaration of Helsinki and all applicable institutional guidelines and regulations.

### TcB measurements

TcB measurements were obtained using the BiliCheck bilirubinometer (SpectRx Inc., Norcross, Georgia, USA) as part of routine clinical care. Measurements were obtained every 12–24 h during the first week of life, with additional measurements performed when clinically indicated or when bilirubin concentrations approached treatment thresholds. TcB measurements were not obtained during active phototherapy, in accordance with the manufacturer’s recommendations.

Because TcB measurements were obtained according to clinical need rather than a fixed research schedule, the timing and number of measurements varied among infants. Each TcB value was paired with the infant’s postnatal age in hours at the time of measurement.

### Time standardization of TcB measurements

To permit comparisons among infants measured at different postnatal ages, TcB values were assigned to one of four predefined age categories, including ≤24 h, 25–48 h, 49–72 h, and ≥73 h. These intervals correspond to clinically meaningful periods of bilirubin production and clearance, allowing comparison of bilirubin concentrations during similar developmental windows.

For evaluation of within-infant bilirubin trajectories, sequential measurements were designated chronologically as S1 through S6. Because individual measurements occurred at variable postnatal ages, sequential observations were used only to examine within-infant trends and calculate kinetic indices and were not interpreted as occurring at uniform ages across infants.

### Bilirubin kinetic indices

Three kinetic indices were calculated from sequential TcB measurements.

Absolute rate of bilirubin increase (RBIa) was defined as:

$${{\rm{RBIa}}}={{\rm{TcB}}}\div{{\rm{postnatal}}}\; {{\rm{age}}}\left({{\rm{mg}}}/{{\rm{dL}}}\; {{\rm{per}}}\; {{\rm{hour}}}\right)$$

This measure reflects the integrated rate of bilirubin accumulation from birth.

Interval rate of bilirubin increase (RBIb) was defined as:

$${{\rm{RBIb}}}=\Delta {{\rm{TcB}}}\div\Delta {{\rm{time}}}\left({{\rm{mg}}}/{{\rm{dL}}}\; {{\rm{per}}}\; {{\rm{hour}}}\right)$$

This measure represents the short-term rate of change between two consecutive measurements. Positive values indicate bilirubin accumulation, and negative values indicate net bilirubin elimination.

Absolute percent change (APC%) was defined as:

$${{\rm{APC}}} \% =\left[\left({{{\rm{TcB}}}}_{2}-{{{\rm{TcB}}}}_{1}\right)\right]\times 100$$

This index expresses the relative change in bilirubin concentration between consecutive measurements and normalizes changes for differences in baseline bilirubin values.

### Phototherapy criteria

Phototherapy was initiated according to institutional guidelines for preterm infants using gestational age-specific treatment thresholds adapted from published recommendations for the management of hyperbilirubinemia in preterm infants [2]. Elevated TcB values prompted confirmatory serum bilirubin measurements, and decisions regarding initiation of phototherapy were based on total serum bilirubin concentrations rather than TcB measurements alone.

### Escalation-of-care threshold

Because validated escalation-of-care thresholds do not exist for infants born before 35 weeks’ gestation, an exploratory surveillance threshold was developed to identify infants approaching treatment criteria, adapted from the American Academy of Pediatrics criteria [5]. This threshold was defined as 3 mg/dL below the gestational age-specific phototherapy threshold and was used solely for hypothesis generation, not as a clinical recommendation.

The distance from this exploratory threshold was expressed as a continuous variable in milligrams per deciliter and was analyzed only in infants who did not receive phototherapy because treated infants had already crossed therapeutic thresholds.

### Statistical analysis

Continuous variables are presented as mean ± standard deviation and categorical variables as frequencies and percentages. Comparisons between infants who did and did not receive phototherapy were performed using Student’s *t* test for continuous variables and the chi-square test or Fisher’s exact test for categorical variables, as appropriate.

Relationships between kinetic variables and peak bilirubin concentrations were assessed using Pearson correlation coefficients.

Multivariable logistic regression was used to determine independent predictors of phototherapy. Candidate predictors included TcB concentration, gestational age, birth weight, and postnatal age measurement. Predictor variables were standardized to permit comparison of effect sizes, and odds ratios were expressed per one standard deviation change.

Multivariable linear regression was used to identify predictors of proximity to the exploratory escalation threshold. Models included gestational age, birth weight, and kinetic indices.

The discriminative ability of the phototherapy prediction model was assessed by calculating the area under the receiver operating characteristic curve, with 95 percent confidence intervals obtained via bootstrap resampling (2000 iterations).

All analyses were performed using R version 4.3 (R Foundation for Statistical Computing, Vienna, Austria). A two-sided *P* value of less than 0.05 was considered statistically significant.

Artificial intelligence tools (Claude, Anthropic) were used solely to assist with language editing and statistical code development. All analyses, interpretations, and final manuscript content were independently reviewed and approved by the authors, who take full responsibility for the work.

## Results

### Population characteristics

The study included 113 preterm infants with a mean gestational age of 32.4 ± 2.9 weeks and a mean birth weight of 1862 ± 656 g (Table 1). Twenty-eight infants (24.8%) received phototherapy, and 85 (75.2%) did not.

**Table 1:** Demographic and clinical characteristics of the study cohort.

Characteristic	No phototherapy (*n* = 85)	Phototherapy (*n* = 28)
Birth weight (g), mean ± SD	1929 ± 626	1659 ± 713*
Gestational age (weeks), mean ± SD	32.9 ± 2.6	31.1 ± 3.3**
GA category, *n* (%)		
<28 weeks	6 (7.1%)	6 (21.4%)
28–31.9 weeks	17 (20.0%)	9 (32.1%)
32–33.9 weeks	23 (27.1%)	5 (17.9%)
34–35.9 weeks	39 (45.9%)	8 (28.6%)
Sex, *n* (%)		
Female	46 (54.1%)	13 (46.4%)
Male	39 (45.9%)	15 (53.6%)
Race/Ethnicity, *n* (%)		
White	33 (38.8%)	11 (39.3%)
Hispanic	26 (30.6%)	8 (28.6%)
Black	17 (20.0%)	4 (14.3%)
Asian	6 (7.1%)	5 (17.9%)
Multiracial/Other	3 (3.5%)	0 (0.0%)
Coombs positive, *n* (%)	3 (3.5%)	1 (3.6%)

^*^p = 0.058; **p = 0.004.

Infants requiring phototherapy were significantly less mature than untreated infants, with a lower mean gestational age (31.1 ± 3.3 versus 32.9 ± 2.6 weeks, *P* = 0.004). Birth weight was also lower among treated infants (1659 ± 713 versus 1929 ± 626 g), although this difference did not reach statistical significance (*P* = 0.058). The phototherapy group included a greater proportion of infants born before 28 weeks’ gestation (21.4% versus 7.1%). There were no significant differences between groups with respect to sex, race and ethnicity, or Coombs positivity.

### TcB according to postnatal age

**Fig. 1:**
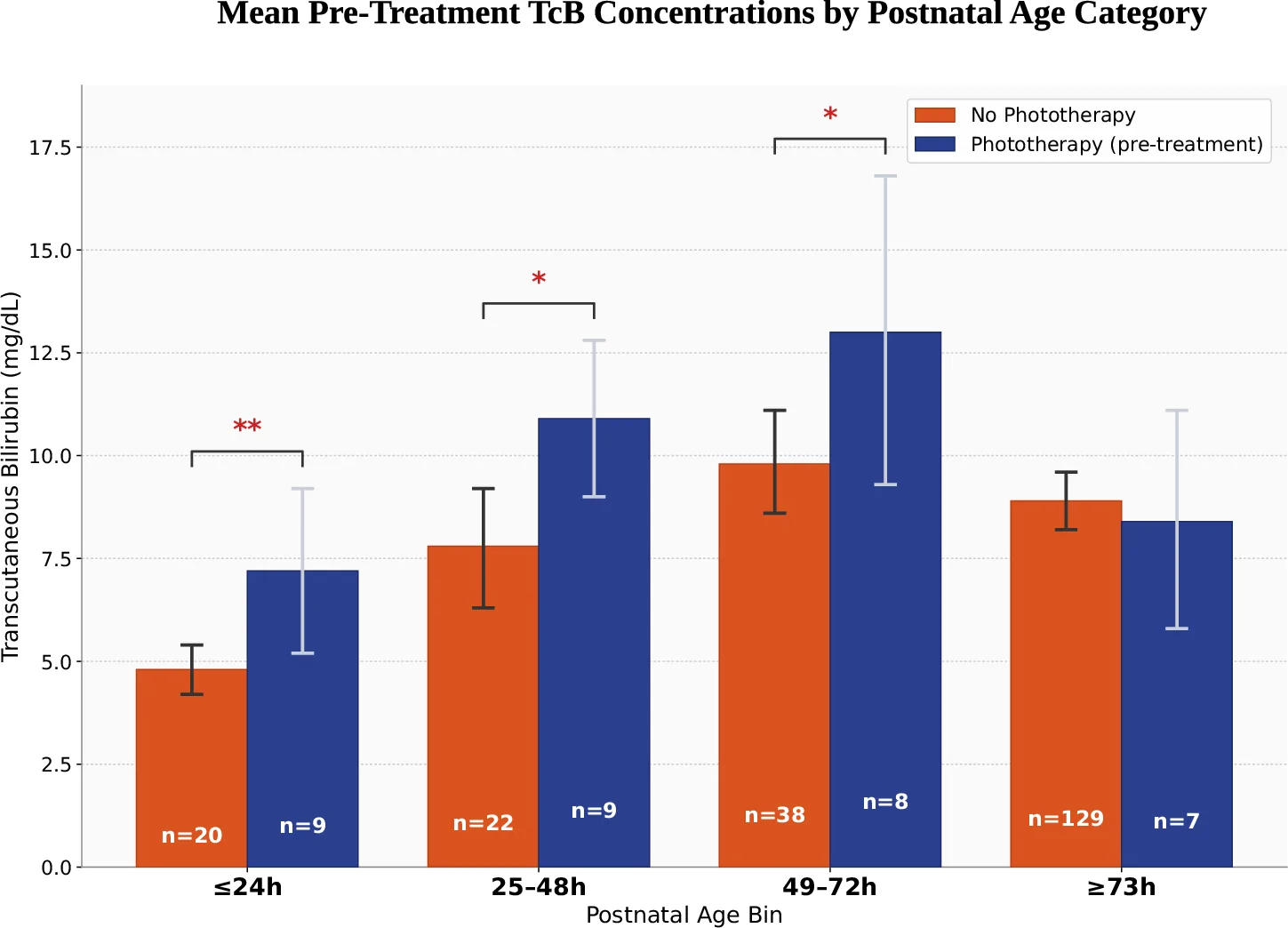
Mean pre-treatment TcB concentrations by postnatal age category. Mean TcB concentrations (±95% confidence intervals) for infants who did and did not receive phototherapy across four standardized postnatal age categories. Only pre-treatment measurements are shown for infants receiving phototherapy.

When TcB values were examined within standardized postnatal age categories, infants who subsequently required phototherapy consistently demonstrated higher bilirubin concentrations during the first 72 h of life (Table 2, Fig. 1).

**Table 2:** Pre-treatment TcB concentrations according to postnatal age and phototherapy status.

Postnatal age bin	No-phototherapy mean ± SD (95% CI) [*n*]	Phototherapy mean ± SD (95% CI) [*n*]	*p*-value
≤24 h	4.81 ± 1.31 (4.20–5.43) [*n* = 20]	7.22 ± 2.58 (5.24–9.21) [*n* = 9]	0.003
25–48 h	7.77 ± 3.33 (6.29–9.25) [*n* = 22]	10.90 ± 2.53 (8.96–12.84) [*n* = 9]	0.007
49–72 h	9.83 ± 3.79 (8.59–11.08) [*n* = 38]	13.03 ± 4.46 (9.30–16.75) [*n* = 8]	0.016
≥73 h	8.96 ± 3.65 (8.32–9.59) [*n* = 129]	8.39 ± 2.87 (5.73–11.04) [*n* = 7]	0.580

Values are mean ± SD with 95% confidence intervals in parentheses. Only pretreatment measurements are included for infants receiving phototherapy.

During the first 24 h, mean TcB values were 7.22 ± 2.58 mg/dL in infants who later received phototherapy compared with 4.81 ± 1.31 mg/dL in untreated infants (*P* = 0.003). The largest difference was observed between 25 and 48 h of age, when TcB values were 10.90 ± 2.53 mg/dL and 7.77 ± 3.33 mg/dL, respectively (*P* = 0.007). The difference persisted during the 49-to-72-h interval (13.03 ± 4.46 versus 9.83 ± 3.79 mg/dL, *P* = 0.016). By 73 h of age and beyond, bilirubin concentrations no longer differed between groups.

**Fig. 2:**
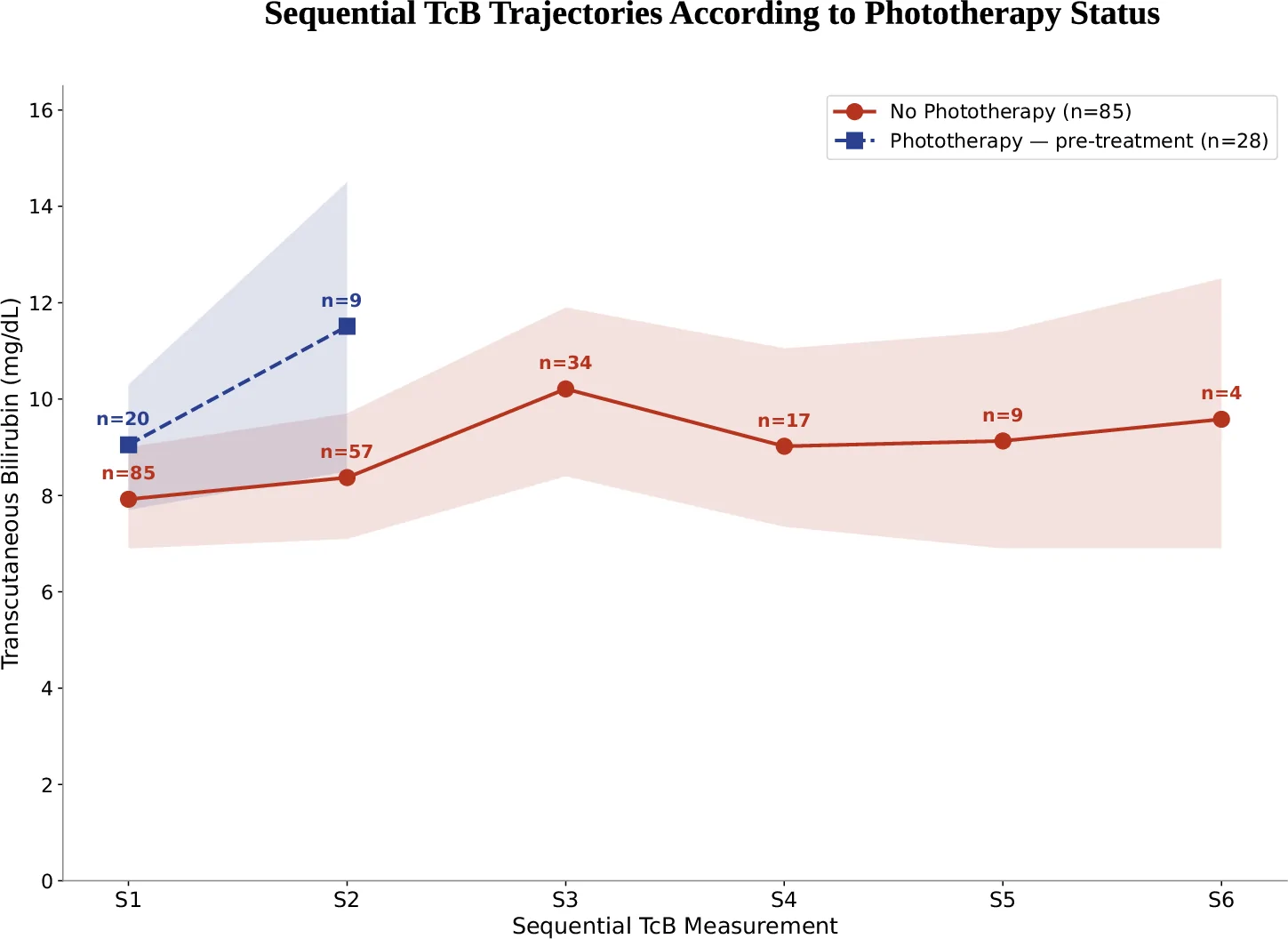
Receiver operating characteristic curve for prediction of phototherapy. Receiver operating characteristic curve for the multivariable logistic regression model incorporating TcB concentration, gestational age, birth weight, and postnatal age. The area under the curve was 0.827 (95% CI, 0.703–0.927).

### Predictors of phototherapy (Fig. 2)

A multivariable logistic regression model incorporating TcB, gestational age, birth weight, and postnatal age demonstrated good discrimination for predicting phototherapy requirements (AUC, 0.827; 95% CI, 0.703–0.927).

After adjusting for covariates, TcB remained an independent predictor of phototherapy. Each one-standard-deviation increase in TcB was associated with a 2.11-fold increase in the odds of subsequent phototherapy. Lower gestational age and earlier postnatal age at measurement were also independently associated with treatment requirement (Table 3).

**Table 3:** Multivariable logistic regression predicting phototherapy requirement.

Predictor variable	Coeff. (β, std.)	Odds ratio (per 1 SD)	Direction	Clinical interpretation
TcB (mg/dL)	0.748	2.11	↑ Risk	Each SD higher TcB doubles phototherapy odds
Gestational age (weeks)	-0.988	0.37	↓ at lower GA	More immature infants at markedly higher risk
Birth weight (g)	0.194	1.21	↑ with higher BW	Partially confounded by GA
Postnatal age at measurement (hours)	-1.193	0.30	↓ with older age	Earlier measurements have highest predictive value
Model AUC (bootstrap 95% CI)	–	–	–	0.827 (0.703–0.927)

Predictors standardized (z-scored).

*OR* odds ratio per 1 SD increase. AUC from 2000 bootstrap samples.

### Bilirubin trajectories and kinetic indices

**Fig. 3:**
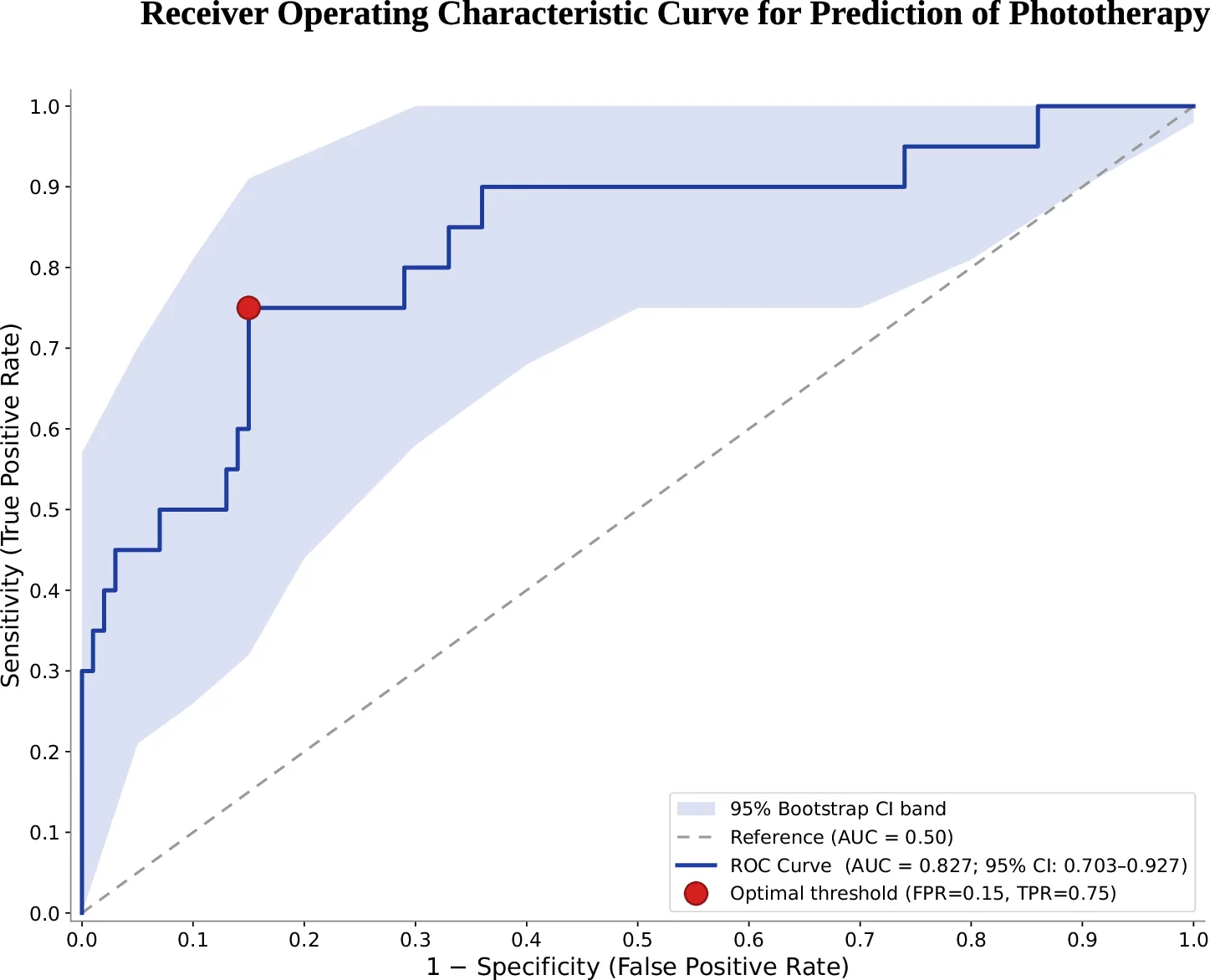
Sequential TcB trajectories according to phototherapy status. Mean TcB concentrations (±95% confidence intervals) at sequential measurements (S1–S6) for infants who did and did not receive phototherapy. Only pre-treatment measurements are shown for treated infants. Sample sizes are indicated at each time point.

Sequential TcB measurements demonstrated distinct bilirubin trajectories between groups (Table 4, Fig. 3). Untreated infants exhibited a typical physiologic pattern, with bilirubin concentrations gradually increasing to a peak at the third measurement and subsequently declining. In contrast, infants requiring phototherapy had higher bilirubin concentrations from the initial measurement and demonstrated a steeper rise before treatment initiation.

**Table 4:** Sequential TcB and kinetic indices.

	No-phototherapy group (*n* = 85)			Phototherapy group (*n* = 28)		
Sample	TcB (mg/dL)	RBIa^a^ (mg/dL/h)	RBIb^b^ (mg/dL/h)	TcB (mg/dL)	RBIa (mg/dL/h)	RBIb (mg/dL/h)
S1	7.9 ± 3.4	0.174 ± 0.132	—	9.0 ± 3.4	0.327 ± 0.175	—
S2	8.4 ± 4.1	0.116 ± 0.079	+0.030 ± 0.132	11.5 ± 4.4	0.240 ± 0.096	+0.144 ± 0.131
S3	10.2 ± 4.1	0.103 ± 0.068	+0.039 ± 0.091	11.2 ± 4.1†	0.126 ± 0.088†	+0.134 ± 0.192
S4	9.0 ± 3.1	0.078 ± 0.038	−0.128 ± 0.241	—	—	—
S5	9.1 ± 3.0	0.068 ± 0.034	+0.018 ± 0.116	—	—	—
S6	9.6 ± 2.8	0.064 ± 0.013	−0.065 ± 0.105	—	—	—

Values are mean ± SD. S1–S6: first through sixth sequential TcB measurement per infant. Pre-treatment data is only for the phototherapy group; groups with *n* < 4 were omitted.

Positive RBIb = net accumulation, negative = net elimination.

^a^RBIa = TcB ÷ postnatal age (mg/dL/h).

^b^RBIb = ΔTcB ÷ Δtime between consecutive measurements (mg/dL/h).

The integrated rate of bilirubin accumulation (RBIa) was significantly higher in infants requiring phototherapy. At the first measurement, RBIa was nearly twice that observed in untreated infants (0.327 ± 0.175 versus 0.174 ± 0.132 mg/dL per hour; *P* < 0.01). Elevated accumulation rates persisted at the second measurement.

The interval rate of bilirubin increase (RBIb) also differed between groups. At the second measurement, the mean rate of bilirubin accumulation was almost five times greater among infants subsequently treated with phototherapy (0.144 ± 0.131 versus 0.030 ± 0.132 mg/dL per hour). In untreated infants, RBIb became negative by the fourth measurement, indicating transition from bilirubin accumulation to net elimination.

Across the entire cohort, early bilirubin accumulation was associated with subsequent bilirubin burden. RBIa, calculated at the first measurement, correlated with the peak pre-treatment TcB concentration (*r* = 0.334, *P* < 0.001).

### Gestational age and exploratory ECT

Among untreated infants, the margin to the exploratory escalation threshold narrowed progressively with advancing postnatal age, indicating increasing proximity to treatment criteria.

In multivariable linear regression analyses adjusted for gestational age and birth weight, absolute percent change at the first measurement independently predicted proximity to the exploratory threshold (β = −0.42; 95% CI, −0.68 to −0.16; *P* = 0.002). Infants with more rapid increases in bilirubin concentration, therefore, approached treatment thresholds more quickly than those with slower bilirubin trajectories.

## Discussion

This study demonstrates that serial transcutaneous bilirubin measurements and dynamic bilirubin kinetic indices can identify preterm infants at increased risk for phototherapy during the first days of life. Infants who required phototherapy had higher TcB concentrations within the first 24 h of life, maintained steeper rates of bilirubin accumulation, and reached treatment thresholds earlier than untreated infants. By restricting analyses to pre-treatment measurements, we characterized native bilirubin kinetics and identified early patterns associated with subsequent therapeutic intervention [12, 13].

The most important finding was the early separation of bilirubin trajectories between groups. During the first 72 h of life, infants requiring phototherapy consistently exhibited higher bilirubin concentrations and greater rates of accumulation than untreated infants. The integrated rate of bilirubin increase from birth (RBIa) was nearly twice as high in infants who required phototherapy, and the short-term interval accumulation rate (RBIb) was almost five times higher during the second measurement interval. Furthermore, early RBIa correlated with the subsequent peak pre-treatment TcB concentration, indicating that the initial trajectory of bilirubin accumulation contains clinically relevant prognostic information. These findings extend the principles of hour-specific bilirubin prediction developed in term infants to the preterm population, in whom reliable prediction tools remain limited [6, 7].

Current management strategies for neonatal hyperbilirubinemia rely primarily on isolated bilirubin measurements and gestational age-specific treatment thresholds. Although these approaches identify infants who have already reached treatment criteria, they provide limited information regarding the trajectory of bilirubin accumulation. Our findings suggest that dynamic measurements, particularly RBIa and APC%, may complement static thresholds by identifying infants whose bilirubin concentrations are rising rapidly despite values that remain below treatment limits. Similar approaches have improved the prediction of significant hyperbilirubinemia in term and late preterm infants and may prove useful in preterm populations as well [6, 8, 15].

Although many extremely premature infants eventually require phototherapy, substantial variability exists in both the timing and magnitude of bilirubin accumulation. Earlier identification of infants at highest risk may facilitate individualized surveillance strategies, optimize the timing of serum bilirubin measurements, and potentially reduce unnecessary laboratory testing. Such an approach may be particularly valuable in preterm infants, in whom bilirubin neurotoxicity can occur at lower total serum bilirubin concentrations than those observed in term newborns [1, 4, 16].

Our findings also underscore the complexity of bilirubin metabolism in preterm infants. Among untreated infants, more advanced gestational age and higher birth weight were associated with higher TcB concentrations later in the neonatal course. This paradoxical relationship reflects the competing influences of bilirubin production and hepatic clearance. More mature preterm infants possess greater erythrocyte mass and increased heme turnover, resulting in larger bilirubin loads, while simultaneously demonstrating improved conjugation and excretory capacity [2, 17]. These observations emphasize that bilirubin metabolism in preterm infants cannot be adequately characterized by a single threshold or by gestational age alone.

Previous studies have demonstrated the reliability of transcutaneous bilirubin measurements in preterm infants and supported their use as a noninvasive screening tool [12, 13]. However, little information is available regarding native bilirubin kinetics and the ability of serial TcB measurements to predict subsequent treatment requirements. Our findings are consistent with reports emphasizing the importance of age-dependent bilirubin trajectories and sequential assessment rather than reliance on isolated measurements [7, 8, 17, 18]. The discriminative performance of our multivariable model, with an area under the receiver operating characteristic curve of 0.827, compares favorably with previously reported prediction models developed in more mature populations [6, 18].

The present study was not designed to evaluate differences in bilirubin kinetics according to race or skin pigmentation and may have been underpowered to detect such effects. However, a previous analysis of this same cohort found no clinically important directional bias related to race or ethnicity in TcB measurements [14]. Additional studies specifically designed to examine the influence of skin pigmentation on bilirubin kinetics and prediction models are warranted.

Several limitations should be considered when interpreting our findings. First, TcB measurements were obtained according to clinical indications rather than at protocol-defined intervals, introducing variability in the timing and number of observations. Second, because most high-risk infants-initiated phototherapy early, fewer pre-treatment measurements were available at later time points in the treated cohort. Third, this was a single-center study with a modest sample size, and external validation in larger multicenter populations is necessary before these findings can be generalized. Fourth, routine screening for glucose-6-phosphate dehydrogenase deficiency was not performed, and therefore its potential contribution to bilirubin kinetics could not be assessed. Finally, the exploratory surveillance threshold, set 3 mg/dL below phototherapy treatment criteria, was developed solely for hypothesis generation because validated escalation-of-care thresholds do not exist for infants born before 35 weeks’ gestation. The clinical utility of this construct requires prospective evaluation before it can be considered routine practice [5].

In conclusion, serial TcB measurements and dynamic bilirubin kinetic indices identify preterm infants at increased risk for phototherapy during the first 24–48 h of life. Early rates of bilirubin accumulation and relative changes in TcB provide predictive information beyond isolated bilirubin measurements and may improve risk stratification in preterm infants. Integration of dynamic kinetic indices with gestational age-specific treatment guidelines may enhance surveillance strategies in the neonatal intensive care unit, although prospective multicenter validation is needed before implementation into clinical practice.

## Conclusions

Serial transcutaneous bilirubin measurements and dynamic bilirubin kinetic indices can identify preterm infants at increased risk for phototherapy during the first 24–48 h of life. Infants who subsequently required treatment demonstrated higher early TcB concentrations and significantly faster rates of bilirubin accumulation than untreated infants. Measures of bilirubin kinetics, including the integrated rate of bilirubin increase and absolute percent change, provided predictive information beyond isolated bilirubin values and gestational age alone.

These findings suggest that incorporation of dynamic, rate-based metrics into existing gestational age-specific management strategies may improve risk stratification and surveillance of hyperbilirubinemia in preterm infants. However, because this was a single-center study and the proposed surveillance threshold was exploratory, prospective validation in larger multicenter cohorts is necessary before these approaches can be recommended for routine clinical use.

## Supplementary information

Checklist

## Data Availability

The data that support the findings of this study are not openly available due to reasons of sensitivity and are available from the corresponding author upon reasonable request.
